# Validation en arabe littéraire chez les militaires tunisiens de la *Post-Traumatic Stress Disorder checklist for DSM-5*

**DOI:** 10.11604/pamj.2022.43.28.35869

**Published:** 2022-09-19

**Authors:** Chaker Bencheikh Brahim, Hamdi El Kefi, Ameni Awissawi, Wafa Kabtni, Abir Baatout, Imen Bouizouita, Chaker Bouguerra, Sami Eddif, Abdelaziz Oumaya

**Affiliations:** 1 Faculté de Médecine de Tunis, Université de Tunis El Manar, Service de Psychiatrie, Hôpital Militaire Principal d´Instruction de Tunis, Tunis, Tunisie,; 2 Unité de Recherche UR17DN05 « Soutien Sanitaire Médico-chirurgical des Forces Armées en Opérations et en Situations d’Exception », Hôpital Militaire Principal d´Instruction de Tunis, Tunis, Tunisie,; 3 Service de Médecine Préventive, Faculté de Médecine de Tunis, Université de Tunis El Manar, Direction Générale de la Santé Militaire, Tunis, Tunisie

**Keywords:** Psychiatrie, échelle, validation, trouble stress post-traumatique, DSM-5, Psychiatry, scale, validation, post-traumatic stress disorder, DSM-5

## Abstract

**Introduction:**

le trouble stress post-traumatique (TSPT) est d´actualité devant la multiplication des conflits armés et des attentats terroristes durant les dernières décennies. L´échelle PCL-5 permet le dépistage et le suivi des patients atteints de TSPT. Le but de notre travail était de valider une version en arabe littéraire de cette échelle sur une population de militaires tunisiens.

**Méthodes:**

la traduction en arabe littéraire tunisien de l´échelle PCL-5 a été réalisée selon la technique de rétro-traduction décrite par Werner et Campbell et recommandée par Brislin. La validation transculturelle a été menée en 7 étapes conformément à la méthode de Vallerand. La collecte des données a été réalisée au Service de Psychiatrie de l´Hôpital Militaire Principal d´Instruction de Tunis (HMPIT) de février 2019 à décembre 2020.

**Résultats:**

nous avons recruté 300 militaires dont 150 suivis pour TSPT. L´indice alpha de Cronbach était de 0,98 ce qui indique une bonne cohérence interne. L´étude des corrélations inter-items a révélé un indice de Spearman total de 0,75. Cet indice indique une bonne homogénéité des items de l´échelle traduite. La validité de construit a été vérifiée à l´aide de l´indice de Kkaiser-Meyer-Olkin (K-M-O) et du test de sphéricité de Bartlett. Ce dernier était significatif (p<0,000) et l´indice de K-M-O était de 0,969, ce qui indique que les corrélations entre les items étaient de bonne qualité.

**Conclusion:**

notre étude a montré que la version arabe de l´échelle PCL-5 a des propriétés psychométriques satisfaisantes comparables à celles de la version originale.

## Abstract (en)

**Introduction:**

post-traumatic stress disorder (PTSD) is a topical issue due to the increase in armed conflicts and terrorist attacks in recent decades. The PCL-5 scale is used to screen and monitor patients with PTSD. The aim of our work was to validate a literary Arabic version of this scale on a population of Tunisian soldiers.

**Methods:**

the translation of the PCL-5 scale into Tunisian literary Arabic was performed according to the back-translation technique described by Werner and Campbell and recommended by Brislin. Cross-cultural validation was conducted in 7 steps according to Vallerand’s method. Data collection was conducted at the psychiatry department of the Military Hospital of Tunis from February 2019 to December 2020.

**Results:**

we recruited 300 military personnel, 150 of whom were followed for PTSD. Cronbach’s alpha was 0.98 indicating good internal consistency. The study of inter-item correlations revealed a total Spearman index of 0.75. This index indicates a good homogeneity of the translated scale items. Construct validity was checked using the Kaiser-Meyer-Olkin index (K-M-O) and Bartlett’s test of sphericity. The latter was significant (p<0.000), and the K-M-O index was 0.969, indicating that the correlations between the items were of good quality.

**Conclusion:**

our study showed that the Arabic version of the PCL-5 scale has satisfactory psychometric properties comparable to those of the original version.

## Introduction

Le Trouble stress post- traumatique (TSPT) est une pathologie psychiatrique aussi vieille que l´humanité [1]. Elle reste d´actualité et a eu un regain d´intérêt ces dernières décennies devant la multiplication des conflits armés et des actes terroristes. Les critères diagnostiques du TSPT selon le DSM-5 se basent principalement sur quatre items ou Clusters. Mis à part de l´item A, qui est l´exposition à un événement traumatisant, s´ajoutent les items B, C, D et E [2].

En premier lieu, l´item B décrit des symptômes envahissants tels que les souvenirs répétitifs involontaires, les rêves répétitifs et le sentiment intense ou prolongé de détresse psychique lors de l´exposition à des indices internes ou externes évoquant l´évènement traumatique en cause [2]. L´item C souligne l´évitement des souvenirs, pensées ou des rappels externes du traumatisme. L´item D représente les altérations négatives des cognitions et de l´humeur comme le sentiment de détachement d´autrui, la réduction nette de l´intérêt pour des activités importantes [2]. Pour finir, l´item E se résume en des altérations marquées de l´éveil, de la réactivité, du sommeil et de la concentration [2].

Pour le diagnostic du TSPT et l'évaluation lors du suivi thérapeutique, le recours à des instruments de mesure est une pratique répandue. Il importe toutefois d'user de ces instruments selon leurs qualités psychométriques démontrées et leur adaptation adéquate pour la langue et la culture dans laquelle le clinicien doit intervenir. Parmi ces échelles, la « Post-traumatic stress disorder Checklist for DSM-5 » qui a été publiée dans sa version originale anglaise en 2013 [3]. La PCL-5 a été ensuite traduite et validée en plusieurs langues [4-9]. Le but de notre étude était de traduire la PCL-5 en arabe littéraire tout en vérifiant sa validité et sa fiabilité au sein d´une population de militaires tunisiens.

## Méthodes

**Cadre et conception de l´étude:** nous avons mené un travail de traduction et de validation psychométrique de l´échelle PCL-5. La traduction en arabe littéraire tunisien a été réalisée selon la technique de rétro-traduction décrite par Werner et Campbell et recommandée par Brislin [10]. La validation transculturelle a été menée en 7 étapes conformément à la méthode de Vallerand (1989). La collecte des données a été réalisée au Service de Psychiatrie de l´Hôpital Militaire Principal d´Instruction de Tunis (HMPIT) de février 2019 à décembre 2020.

**Population étudiée:** la taille de notre échantillon a été calculée selon la règle de Nunnally qui recommande d´avoir un nombre de sujets égal au moins à dix fois le nombre de questions [11]. Tenant compte de cette règle statistique nous avons estimé la taille minimale de l´échantillon à 200 participants. Au total 300 fiches de recueil de données ont été distribuées. La population cible était constituée de 150 militaires suivis au service de psychiatrie de l´HMPIT pour un TSPT et 150 militaires non atteints de TSPT. Nous avons opté pour les critères d´inclusion suivants: âge supérieur à 18 ans, maitrise de la langue arabe, acceptation de participer à l´étude et absence de troubles psychiatriques graves altérant les capacités de jugement. Une réponse incomplète au questionnaire était considérée comme critère d´exclusion.

**Recueil des données:** le recueil des données a été réalisé par des psychiatres et des psychologues du Service de Psychiatrie de l´HMPIT. Après un bref entretien initial permettant de vérifier les critères d´inclusion, chaque participant a reçu la fiche de recueil des données sociodémographiques et la version de l´échelle PCL-5 traduite en arabe. Les fiches étaient récupérées par l´enquêteur immédiatement après leur remplissage.

### Traduction et processus de validation

Une première traduction du questionnaire a été effectuée de la langue source anglaise à l´arabe littéraire par deux traducteurs bilingues. Après une réunion rassemblant les traducteurs et les experts, un consensus a été trouvé sur une première version préliminaire en arabe littéraire. Cette version préliminaire a été par la suite rétro-traduite en anglais par deux autres traducteurs bilingues. La version originale en anglais et la version obtenue par rétro-traduction ont été contrôlées au niveau de l´adéquation de traduction par les experts. A la fin de cette troisième étape, nous avons obtenu une version expérimentale de la PCL-5 en arabe littéraire. L´équipe d´experts étaient composée de six psychiatres et de deux psychologues.

Le terme « validité » se rapporte à la capacité d'un instrument de mesure d´évaluer finement ce pour quoi il a été créé [12]. Pour vérifier la validité de la version traduite de la PCL-5, nous avons testé la validité de contenu et la validité de construit. Initialement l´équipe d´experts s´est prononcée sur la clarté, la pertinence et la capacité de discrimination de chacun des items. Pour chaque item, il a été demandé de noter chacun des trois paramètres selon une échelle ordinale de 1 à 4 (1) très faible; 2) faible; 3) élevée; 4) très élevée). Les items qui ont obtenu un score de 2 ou moins ont été modifiés conformément aux recommandations des experts.

Secondairement un pré-test a été mené auprès de 20 sujets choisis aléatoirement. Nous avons demandé à ces participants de répondre au questionnaire traduit et de noter la clarté de chaque énoncé de l´instrument sur une échelle de Likert allant de 1 (pas clair du tout) à 5 (très clair) et de repérer les questions qui peuvent induire une gêne. Les éléments jugés ambigus au pré-test ont été examinés par le comité d´experts, puis remplacés par des éléments porteurs du sens désiré. Les items notés de 2 ou moins sur l´échelle de clarté désignaient les éléments à modifier. Au terme de cette étape, nous avons obtenu une version finale en arabe littéraire de la PCL-5. Enfin nous avons évalué la fidélité du questionnaire par le calcul du coefficient alpha de Cronbach, la corrélation par le calcul du coefficient de Spearman (r) et la validité de structure interne par l´analyse factorielle exploratoire (AFE). Nous n´avons pas procédé à l'étude de la stabilité temporelle et à la mesure de la reproductibilité par l´administration du questionnaire une seconde fois (test-retest) à un groupe de participants.

### Analyse statistique

La saisie et l´analyse des données ont été réalisées à l´aide du logiciel statistique *Statistical Package for the Social Sciences (SPSS)* version 21.0. Nous avons calculé le coefficient alpha qui permet de mesurer la cohérence interne du questionnaire, et qui évalue l'homogénéité des items dans le questionnaire. Il vérifie que les mesures répétées à l´intérieur d´un même questionnaire sont convergentes [13]. Le coefficient alpha de Cronbach peut varier de 0 à 1 [13]. Une valeur de 0,5 est acceptable et des valeurs allant de 0,70 à 0,85 sont souhaitables [14].

Le coefficient de Spearman (r) a été utilisé dans notre étude pour apprécier la corrélation inter-items [15]. La force de la corrélation inter-items peut être interprétée comme suit: corrélation très faible si 0,00 ≤ r < 0,20; corrélation faible si 0,20 ≤ r < 0,40; corrélation modérée si 0,40≤r<0,60; corrélation forte si 0,60 ≤ r < 0,80; corrélation très forte si 0,80 < r < 1 [16]. Le nombre de facteurs extraits de la matrice des corrélations a été identifié en utilisant le critère de la « valeur propre initiale ». Par convention, tout facteur avec une valeur propre initiale supérieure ou égale à 1 est considéré comme facteur significatif [15]. Seuls les items dont les saturations étaient supérieures à 0,05 ont été retenus.

La mesure d´adéquation de l´échantillonnage de Kaiser-Meyer-Olkin (K-M-O) permet d´évaluer dans quelle mesure l´ensemble des variables sélectionnées est un ensemble cohérent qui permet de définir une solution pertinente en termes conceptuels [17]. Les statistiques se calculent aussi bien pour l´ensemble de la matrice des corrélations que pour chacune des variables et prennent des valeurs dans l´échelle de 0 à 1 [18]. Une variable pertinente pour l'analyse devrait obtenir un K-M-O supérieur à 0,5. Le test de sphéricité de Bartlett vérifie l'hypothèse nulle selon laquelle toutes les corrélations seraient égales à zéro [19]. Nous devons rejeter l'hypothèse nulle afin que le test soit significatif. Pour cela, la probabilité d'obtenir la valeur du test doit être plus petite que 5% [19]. Seuls les items définissant l´état de stress post- traumatique ont été soumis à cette validation. Le seuil de signification a été fixé à p ≤ 5%.

**Considérations éthiques:** le protocole de l´étude a été approuvé par le Comité Local de Protection des Personnes de l´HMPIT en date du 10/12/2018 (référence: 16/2018/CLPP-HMPIT). L´accord de l´équipe ayant mis au point la version originale de la PCL-5 a été obtenu pour la traduction et l´utilisation de l´échelle bien qu´elle appartienne au domaine public.

## Résultats

### Données démographiques et cliniques de la population étudiée

L´âge moyen des répondants était de 32 ans avec des âges extrêmes allant de 21 à 48 ans. Notre échantillon se composait de 97,3% d´hommes (n=292) et seulement 2,7% de femmes (n=8). Cent cinquante-six participants (52,7%) avaient une origine urbaine, alors que 47,3% (n=144) avaient une origine rurale. Quatre-vingt-dix-huit participants (32,77 %) étaient mariés, 44,93% célibataires (n=125), 20,95% fiancés (n=63) et 1,4% veufs ou divorcés (n=4). Notre échantillon ne comportait que des individus scolarisés.

### Score global de la PCL-5

La moyenne calculée du score global de la PCL-5 était de 42,92 ± 24,2 avec des extrêmes de 5 et de 74.

### Validité du contenu

Les experts ont examiné et vérifié la clarté, la pertinence et la capacité de discrimination de chaque item. Une clarté satisfaisante a été confirmée pour 75% des items, la pertinence a été jugée bonne pour 85% des items et 75% des énoncés étaient de bonne valeur discriminative. La clarté des items 6, 7, 14, 16 et 17 a été critiquée par les experts. Les items 12,17 et 18 ont été jugés initialement peu pertinents et les items 3, 8, 9 et 12 manquaient de pouvoir discriminatif. Ces critiques ont permis d´améliorer la formulation des items pour les rendre plus compréhensibles. Les participants au prétest se sont exprimés sur la clarté des items, dont 81% ont été notés entre 3 et 5. Les items 7, 17 et 18 ont obtenu les notes les plus faibles et ont été reformulés. Le coefficient alpha de Cronbach pour les 20 items était de 0,98. L´étude du coefficient alpha si critère supprimé a montré que, si on en retire un, le coefficient alpha de Cronbach varie **faiblement (Tableau 1**).

**Tableau 1:** coefficient alpha de Cronbach si item supprimé

Items	Coefficient alpha si item supprimé
Item 1	0,981
Item 2	0,981
Item 3	0,981
Item 4	0,982
Item 5	0,981
Item 6	0,982
Item 7	0,982
Item 8	0,981
Item 9	0,981
Item 10	0,982
Item 11	0,981
Item 12	0,981
Item 13	0,981
Item 14	0,981
Item 15	0,981
Item 16	0,981
Item 17	0,981
Item 18	0,981
Item 19	0,982
Item 20	0,983

### Validité de construction

L´indice de K-M-O dans notre étude était de 0,969. Le résultat du test de sphéricité de Bartlett était significatif (p<0,000) permettant de poursuivre l´analyse factorielle. L´analyse factorielle exploratoire après rotation Varimax a fait dégager sept facteurs (ou composants). Ces facteurs étaient capables d´expliquer 89% de la variance totale. Les trois premiers facteurs étaient particulièrement responsables de 48,1% de la variance totale expliquée. Les facteurs ont été par la suite classés en plusieurs catégories (Tableau 2). Le Tableau 3 résume la matrice de variance de ces différents facteurs.

**Tableau 2:** variance des composants après rotation Varimax

Facteurs	Items	Catégories	Valeur après rotation	Pourcentage de la Variance
Facteur 1	1, 4, 6, 14 et 16	Reviviscence (R)	3,63	18,18
Facteur 2	2, 7, 8 et 9	Affectes Négatifs (AN)	3,25	16,26
Facteur 3	3, 12, 15 et 17	Comportement d´extériorisation (CE)	2,72	13,61
Facteur 4	5, 11 et 18	Eveil anxieux (EA)	2,71	13,56
Facteur 5	19	Dysphorie (D)	2,07	10,38
Facteur 6	13 et 20	Anhédonie (A)	1,94	9,73
Facteur 7	10	Evitement (E)	1,47	7,39

**Tableau 3:** matrice des facteurs après rotation Varimax

	Facteur 1	Facteur 2	Facteur 3	Facteur 4	Facteur 5	Facteur 6	Facteur 7
**Item 1**	0,529						
**Item 2**		0,502					
**Item 3**			0,544				
**Item 4**	0,678						
**Item 5**				0,498			
**Item 6**	0,806						
**Item 7**		0,699					
**Item 8**		0,632					
**Item 9**		0,624					
**Item 10**							0,734
**Item 11**				0,646			
**Item 12**			0,502				
**Item 13**						0,467	
**Item 14**	0,485						
**Item 15**			0,550				
**Item 16**	0,513						
**Item 17**			0,583				
**Item 18**				0,578			
**Item 19**					0,809		
**Item 20**						0,868	

### Corrélations inter-critères

Les résultats ont montré que la répétition (critère B) avait une forte corrélation avec l´altération négative des cognitions (critère D) et l´hyperactivité neurovégétative (critère E). Les coefficients de Spearman étaient respectivement r = 0,798 et r = 0,797. Une très forte corrélation a été notée aussi entre les critères D et E (r =0,898). Le Tableau 4 présente la matrice des corrélations inter-critères.

**Tableau 4:** matrice des corrélations inter-critères

	Critère B	Critère C	Critère D	Critère E
Critère B	1,000			
Critère C	0,825	1,000		
Critère D	0,798	0,808	1,000	
Critère E	0,797	0,785	0,898	1,000

### Corrélations inter-items

Certains items avaient une corrélation modérée, ce qui était le cas entre l´item 6 et l´item 20 (r= 0,522). D´autres étaient fortement corrélés, comme les items 8 et 13 (r= 0,771). La corrélation des items de notre échelle était parfois très forte. On prend l´exemple de l´item 11 et l´item 18, qui avaient un coefficient de Spearman r =0,906. La corrélation moyenne de tous les items était égale à 0,75.

## Discussion

Dans notre étude, la version arabe de l´échelle PCL-5 obtenue par la méthode de traduction et rétro-traduction a eu une bonne acceptabilité au prétest. Concernant la fiabilité, l´indice alpha de Cronbach était de 0,98 ce qui indique une bonne cohérence interne. Cette cohérence a été confirmée davantage en calculant le coefficient de Cronbach après avoir supprimé un item de l´échelle. Ce dernier était stable et très proche du coefficient alpha trouvé pour l´ensemble des items (Î±=0,98), ce qui est témoin d´une bonne consistance inter-items.

L´étude des corrélations inter-items a révélé un indice de Spearman total de 0,75. Cet indice indique une bonne homogénéité des items de l´échelle traduite. La validité de construit a été vérifiée à l´aide de l´indice K-M-O et du test de sphéricité de Bartlett. Ce dernier était significatif et l´indice de K-M-O était de 0,969. Ce qui indique que les corrélations entre les items sont de bonne qualité.

Nos participants appartenaient à l´institution militaire composée essentiellement de sujets jeunes et de sexe masculin. Ce profil pourrait être plus exposé aux événements traumatiques. Il en découle généralement une augmentation des différents scores de sévérité de la PCL-5 par rapport à la population générale [20,21]. Des études futures, basées sur des échantillons représentatifs de la population générale permettraient de généraliser les résultats obtenus lors de la présente recherche sur la population tunisienne. Cependant, les critères stricts d'inclusion et de non-inclusion ont permis de retenir des participants qui présentaient les caractéristiques de la population militaire tunisienne cible. La stabilité temporelle n´a pas pu être vérifiée, des test-retest devront être effectués dans des études ultérieures pour vérifier cette stabilité.

Dans notre étude, la valeur de l´alpha de Cronbach était de 0,98. Ce coefficient est qualifié d´élevé [22]. En effet, la plupart des auteurs définissent une bonne fiabilité par une valeur de l´indice alpha de Cronbach variant entre 0,7 et 0,95 [22]. Notre valeur semble dépasser cet intervalle en se rapprochant de 1. Ce qui peut être considéré par certains auteurs comme très élevée pouvant indiquer une redondance de certains items [22]. En revanche, d´autres qualifient cette valeur d´excellente reflétant l´homogénéité de l´ensemble des items [15]. En revanche, en se référant aux différentes études psychométriques et de validation du PCL-5, on remarque que cette échelle se caractérise par une consistance interne élevée supérieure à 0,9 [4,6,20,23].

L´étude de la cohérence entre les différents critères B, C, D et E a montré une excellente corrélation entre eux (coefficient alpha supérieur à 0,76). Cette valeur tend à diminuer si on supprime l´un des critères, ce qui est un bon indicateur de la consistance de l´échelle. Ces résultats semblent être en harmonie avec les différentes études de validation de la PCl-5. En effet, Ashbaugh *et al*., en validant les deux versions anglaise et française au Canada, ont trouvé une excellente cohérence entre les différents critères B, C, D et E pour les deux échelles [6,7]. Pour étudier les relations inter-items, nous avons estimé leurs consistances en supprimant un item à chaque fois, puis en étudiant son effet sur le coefficient alpha total de l´échelle. Les coefficients trouvés étaient très proches du coefficient alpha pour l´ensemble des items (α=0,98). Ainsi le coefficient alpha si item supprimé a montré une bonne consistance. D´autres études ont calculé le coefficient alpha de Cronbach de chaque item séparément. L´étude allemande de Krüger *et al*. a trouvé que tous les items avaient un coefficient supérieur à 0,74 [4]. Les items constituant le critère B (la répétition) avaient entre 0,77 et 0,92, ceux de l´évitement entre 0,74 et 0,92, ceux de l´altération négative des cognitions entre 0,78 et 0,89 et ceux de l´hyperactivité neurovégétative entre 0,75 et 0,84 [4].

Tous les Clusters avaient des coefficients de Spearman r > 0,7 ce qui témoigne de la forte corrélation entre eux. Il existait une forte corrélation entre l´altération négative des cognitions (critère D) et l´hyperactivité neuro-végétative (critère E). Ces derniers avaient un coefficient de Spearman (r) à 0,89 ce qui peut être considéré par certains auteurs comme un facteur de redondance [24]. Ce comportement a été par ailleurs noté aussi dans l´étude de Pereira-Lima *et al*. [9]. Cette étude faite sur l´échelle PCL-5 traduite en portugais a révélé une très forte corrélation entre ces deux critères en particulier (r=0.88).

L´examen de la matrice des corrélations inter-items, a mis en évidence des corrélations modérées, fortes et très fortes. En effet, les différents coefficients de Spearman variaient de 0,5 à 0,9 ce qui reflète une bonne homogénéité entre les différents items. Les items 11 et 18 avaient un très fort coefficient de Spearman (r =0,906). Ce chiffre suggère que ces items sont très proches au point d´être répétitifs [24]. Ceci peut être expliqué par la relation entre l´altération négative des cognitions et l´hyperactivité neurovégétative.

L´étude des propriétés psychométriques des différentes échelles de PCL-5 a montré que les items sont presque toujours fortement corrélés [8,20]. Ces résultats témoignent de la bonne adaptation de cette échelle aux particularités culturelles de chaque pays. Bien que certains concepts puissent être moins pertinents dans notre contexte culturel, la PCL-5 traduite en arabe littéraire parait fiable linguistiquement. Par ailleurs, la corrélation moyenne de tous les items était à 0,75. Ce chiffre semble être en harmonie avec les autres études et indique une bonne homogénéité des items de l´échelle [8,20].

Dans notre travail, nous nous sommes basés sur le critère de Kaiser. Ainsi, les sept facteurs retenus étaient respectivement: reviviscence, affectes négatifs, comportement d´extériorisation, éveil anxieux, dysphorie, anhédonie et évitement. Ces facteurs expliquaient 89% de la variance totale. Cependant, cette structure ne suit pas le même modèle utilisé dans la version originale de la PCL-5 par Blevins *et al*. [23].

En effet, la PCL a subi une modification factorielle avec le passage du DSM-4 vers leDSM-5. Elle est passée d´un modèle contenant trois facteurs: la reviviscence, l´évitement avec l´engourdissement émotionnel et l´hyperréactivité [25] à un modèle divisé en quatre facteurs. Le modèle DSM-5 a été initialement élaboré par King *et al*. nen 1998 [26] puis modifié par Simms *et al*. [27] pour être finalement adopté en 2013 par Weathers *et al*.dans laPCL-5 [23]. Ce modèle comporte quatre facteurs qui sont en harmonie avec les différents critères diagnostiques du DSM-5. Mais le principal changement concerne le deuxième facteur qui a été divisé en deux: « l´évitement » et « les altérations négatives persistantes dans les cognitions et l'humeur » [23].

Dans la littérature, il existe plusieurs modèles pour la PCL-5. Le modèle « dysphorie » initialement élaboré par Elhai *et al*. en 2011 comporte cinq facteurs [28]. Ce dernier divise les symptômes liés à l´hyperréactivité en deux facteurs différents: l´éveil dysphorique et l´éveil anxieux. Le modèle « anhédonie » à six facteurs respecte la répartition factorielle du « dysphorie » mais divise les symptômes liés à l´altération négative de l´humeur et des cognitions entre deux facteurs séparés en anhédonie et affectes négatifs [29]. Le modèle « hybride » à six facteurs a rattaché les items 15 et 16 à un facteur appelé comportement d´extériorisation au lieu de l´hyperréactivité tout en respectant la répartition factorielle du modèle « Anhédonie » [30]. Pour plusieurs auteurs, ce modèle à six facteurs est le plus adapté à la structure de la PCL-5 [4,6,28,30]. Aucun modèle n´a pu obtenir l´unanimité des auteurs puisqu´il faut choisir le même nombre de participants ayant eu le même évènement traumatisant dans les mêmes circonstances [28]. La validité de construit semble être affectée par le contexte culturel aussi [15]. Par conséquence on ne peut que retenir la propriété multifactorielle de la PCL-5 sans affirmer un nombre précis de facteurs.

## Conclusion

Notre étude a montré que la version arabe de l´échelle PCL-5 a des propriétés psychométriques satisfaisantes comparables à celles de la version originale. Cet instrument est prêt à être utilisé pour le dépistage et le suivi des militaires tunisiens atteints de TSPT.

### Etat des connaissances sur le sujet

- L´échelle PCL-5 est facile d´utilisation pour le dépistage et le suivi des patients atteints de TSPT;
- L´échelle originale en anglais a été traduite en plusieurs langues;
- La validation d´une version en arabe littéraire de cette échelle est nécessaire dans un but clinique et de recherche.

### Contribution de notre étude à la connaissance

- Il s´agit de la première étude en Tunisie qui a traduit en arabe littéraire et évalué les propriétés psychométriques de l´échelle PCL-5;
- Cet instrument peut être utilisé en consultation de psychiatrie militaire en Tunisie et dans les pays nord-africains utilisant un arabe littéraire proche de celui des tunisiens;
- Plusieurs travaux de recherche utilisant cette version de la PCL-5 pourront être menés sur des militaires nord-africains souffrant de TSPT.
